# Checkpoint Kinase 1 Pharmacological Inhibition Synergizes with DNA-Damaging Agents and Overcomes Platinum Resistance in Basal-Like Breast Cancer

**DOI:** 10.3390/ijms21239034

**Published:** 2020-11-27

**Authors:** Cristina Nieto-Jimenez, Ana Alcaraz-Sanabria, Sandra Martinez-Canales, Veronica Corrales-Sanchez, Juan Carlos Montero, Miguel Burgos, Miriam Nuncia-Cantarero, Atanasio Pandiella, Eva M. Galan-Moya, Alberto Ocaña

**Affiliations:** 1 Translational Research Unit and CIBERONC, Albacete University Hospital, 02002 Albacete, Spain; cnjimenez92@gmail.com (C.N.-J.); analucia_alcaraz@hotmail.com (A.A.-S.); sanmarca16@gmail.com (S.M.-C.); vero_nika.s@hotmail.com (V.C.-S.); mburgoslozano@sescam.jccm.es (M.B.); 2 Translational Oncology Laboratory, Centro Regional de Investigaciones Biomédicas (CRIB) and Nursery School, Campus de Albacete, Universidad de Castilla-La Mancha, 02008 Albacete, Spain; miriam_nuncia@hotmail.com; 3 Instituto de Biología Molecular y Celular del Cáncer del CSIC, IBSAL and CIBERONC, 37007 Salamanca, Spain; jcmon@usal.es (J.C.M.); atanasio@usal.es (A.P.); 4 Hospital Clínico Universitario San Carlos, IDISSC and CIBERONC, 28040 Madrid, Spain

**Keywords:** breast cancer, standard-of-care chemotherapies, chemotherapy resistance, DNA damage response (DDR), CHK1 inhibitors, DNA-damaging agents, platinum compounds, synthetic lethality

## Abstract

Basal-like breast cancer is an incurable disease with limited therapeutic options, mainly due to the frequent development of anti-cancer drug resistance. Therefore, identification of druggable targets to improve current therapies and overcome these resistances is a major goal. Targeting DNA repair mechanisms has reached the clinical setting and several strategies, like the inhibition of the CHK1 kinase, are currently in clinical development. Here, using a panel of basal-like cancer cell lines, we explored the synergistic interactions of CHK1 inhibitors (rabusertib and SAR020106) with approved therapies in breast cancer and evaluated their potential to overcome resistance. We identified a synergistic action of these inhibitors with agents that produce DNA damage, like platinum compounds, gemcitabine, and the PARP inhibitor olaparib. Our results demonstrated that the combination of rabusertib with these chemotherapies also has a synergistic impact on tumor initiation, invasion capabilities, and apoptosis in vitro. We also revealed a biochemical effect on DNA damage and caspase-dependent apoptosis pathways through the phosphorylation of H2AX, the degradation of full-length PARP, and the increase of caspases 3 and 8 activity. This agent also demonstrated synergistic activity in a platinum-resistant cell line, inducing an increase in cell death in response to cisplatin only when combined with rabusertib, while no toxic effect was found on non-tumorigenic breast tissue-derived cell lines. Lastly, the combination of CHK1 inhibitor with cisplatin and gemcitabine resulted in more activity than single or double combinations, leading to a higher apoptotic effect. In conclusion, in our study we identify therapeutic options for the clinical development of CHK1 inhibitors, and confirm that the inhibition of this kinase can overcome acquired resistance to cisplatin.

## 1. Introduction

The treatment of solid tumors is still based mainly on chemotherapy [1]. However, the recent identification of molecular alterations in tumors has permitted the design and development of therapies against specific oncogenes [1,2]. This has been the case with the identification of HER2 amplification in breast cancer, which led to the design of antibodies or small tyrosine kinase inhibitors against this protein [3,4]. This approach can be clinically beneficial if the molecular alteration is an oncogenic driver and the experimental agent is able to efficiently act on the target. In line with this, other investigational compounds have reached the clinical setting, including B-RAF or MEK inhibitors in BRAF (V600E)-mutant tumors such as melanoma or non-small cell lung cancer [5,6].

Targeted therapies can also be designed against proteins or pathways that can establish a synthetic lethality interaction when combined with other agents [7,8]. The inhibition of a single pathway causes cells to be more dependent on other routes to maintain cell survival; therefore, those survival routes are optimal targets for drug inhibition [8]. As redundancy in biological systems is a common way to maintain cell homeostasis and protect cells from external insults, the inhibition of two complementary pathways can have a substantial impact on cell death [9]. 

Basal-like breast cancer, which includes the triple negative breast cancer subtype (TNBC), is considered an unsolved medical challenge as patient outcome in the advanced stages is short, even after receiving appropriate treatment [10]. These tumors are characterized by a high grade of genetic instability and a notable impairment of DNA repair processes [1,11] In this context, DNA-damaging agents like platinum compounds have been positioned as key therapies for the treatment of basal-like tumors. However, although these compounds have shown activity, resistance to these agents appears after a given period of time. 

Control of DNA damage is a complex procedure that involves different mechanisms [12]. Multiple players are implicated in the DNA damage response (DDR). The serine/threonine kinase CHK1 is required for checkpoint mediated cell cycle arrest integrating signals from ATM and ATR [13,14,15,16]. CHK1 detects cells with DNA damage, and triggers the activation of mechanisms of DNA repair causing cell arrest in G1 [14,15]. Inactivation of CHK1 permits cell cycle progression without a proper DNA repair, therefore increasing genetic instability and aneuploidy, which may finally lead to cell death [14,15]. For this reason, these DDR inhibitors are promising candidates in the treatment of solid tumors. Indeed, CHK1 inhibitors are currently in clinical development, with several phase I studies exploring the safety of these compounds alone or in combination with different chemotherapies [17,18]. Thus, the identification of chemotherapies that could be used as clinical partners to potentiate their effect is a primary goal. Finally, the identification of druggable mechanisms that can overcome resistance to standard-of-care treatments could open avenues to optimize therapeutic strategies in these indications. 

Therefore, in this study we evaluated the synthetic lethality interactions between CHK1 inhibitors and different chemotherapeutic agents used in the treatment of patients with basal-like breast cancer. We also evaluated the potential of these compounds to overcome resistance to platinum agents.

## 2. Results

### 2.1. Synergistic Interactions of CHK1 Inhibitors with Standard-of-Care Therapies

**Figure 1:**
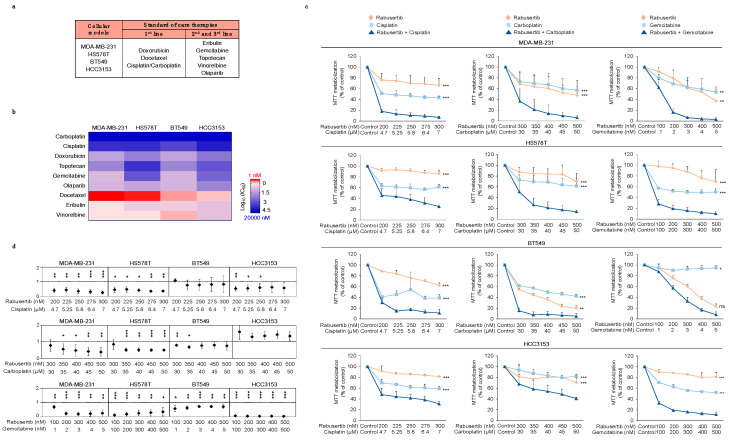
Pharmacological screening of approved therapies in breast cancer. (**a**) The table shows the chemotherapeutic agents currently used for metastatic breast cancer. The cellular models used in the study are also displayed. (**b**) Heat map representing color-coded sensibility levels of breast cancer cell lines to chemotherapeutic agents. Cells were seeded and treated with chemotherapeutic agents for 72 h; then, the IC50 values (logarithmic scale) were defined. (**c**,**d**) Synergistic effect of the CHK1 inhibitor (rabusertib) and platinum-derived compounds and gemcitabine in TNBC cells. Cells were treated at the indicated doses for 72 h. Then, metabolization of MTT in viable cells was determined by spectrophotometry and synergistic effects were analyzed using the CalcuSyn program. CI < 1 = synergistic effect; CI = 1 = additive effect; CI > 1 = antagonistic effect. ns > 0.05, *p* < 0.05 *, *p* < 0.01 **, *p* < 0.001 ***.

To identify the synergistic interactions of standard-of-care therapies with CHK1 inhibitors, we evaluated growth inhibition by measuring MTTs, using currently approved therapies for breast cancer (Figure 1a). We selected agents with different mechanisms of action, including those that inhibit the formation of the mitotic spindle, like vinorelbine, docetaxel, or eribulin, and DNA-damaging agents, such as doxorubicin, platinum compounds (cisplatin and carboplatin), gemcitabine, or topotecan. We also included olaparib, a PARP inhibitor that has recently been approved for the treatment of breast cancers with germ line mutations of the BRCA gene [19]. The IC50 values of these compounds in four representative cell lines of basal-like breast cancer, MDA-MB-231, HS578T, BT549, and HCC3153 are shown in Figure 1b and Figure S1a. Antimitotic agents displayed the greatest antiproliferative activity.

The above standard-of-care drugs were combined with the CHK1 inhibitor rabusertib, an agent that is currently in clinical development. To explore whether the administration of rabusertib was synergistic with any of the chemotherapies mentioned, we used the Chou and Talalay method [20,21]. The IC50 for this compound in all cell lines is shown in Figure S1b. The combination of platinum agents, both cisplatin and carboplatin, and gemcitabine with the CHK1 inhibitor rabusertib showed a synergistic anti-proliferative effect in most of the cell lines tested (Figure 1c,d). This effect was not observed on the non-transformed epithelial cell line MCF10A and the fibroblasts CRL-2072, derived from normal breast tissue from the mammary gland and the skin, respectively (Figure S2). When rabusertib was combined with the PARP inhibitor olaparib, only MDA-MB-231 and HCC3153 displayed a clear synergistic response (Figure S3). 

Doxorubicin, another DNA-damaging agent, showed less activity in the breast models than the previous DNA-targeting compounds, while combinations with topotecan appeared to be highly synergistic for most of the cell lines (Figure S4a).

In sharp contrast, the combination of rabusertib with agents that target mitosis, like vinorelbine, docetaxel, and eribulin were not synergistic at any of the evaluated doses (Figure S4b). 

Given the high synergistic effect displayed by platinum compounds with rabusertib, we also explored the effect of the combination of these therapies with another CHK1 inhibitor also in clinical development, SAR020106. The synergistic interaction found for both platinum compounds and rabusertib was also confirmed with SAR020106 (Figure S5).

Altogether, these results demonstrate that the inhibition of CHK1 has a strong synergistic interaction with DNA-damaging agents, mainly platinum compounds but also gemcitabine, topotecan, and the novel PARP inhibitor olaparib on basal-like cancer cell lines. 

### 2.2. CHK1 Inhibition Reduces Cell Growth in Combination with Platinum Compounds

**Figure 2:**
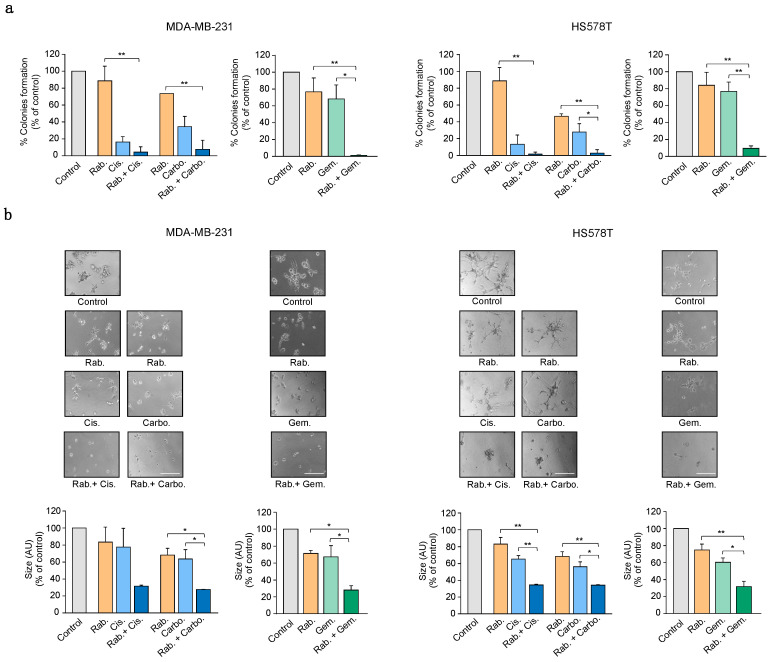
The combination of CHK1 inhibitor with platinum derivates or gemcitabine affects colony formation and invasiveness. (**a**) Colony formation assays in MDA-MB-231 and HS578T. Cell were treated for 24 h with rabusertib (Rab. 300 nM or 350 nM for cisplatin and gemcitabine or carboplatin sample sets, respectively) alone or in combination with cisplatin (Cis. 7 μM), carboplatin (Carbo. 35 μM), or gemcitabine (3 nM for MDA-MB-231 and 300nM for HS578T). Percentage of colonies referred to non-treated controls is shown. (**b**) Matrigel invasion assays in TNBC cell lines. Cells were seeded on Matrigel-coated wells, and then treated with the drugs at the same doses as in (**a**) for 48 h. The formation of 3D structures was evaluated by microscopy. Scale bar = 200 μM. (**a**,**b**) Statistics of single against double combination are shown. *p* < 0.05 *, *p* < 0.01 **.

To evaluate the long-term effect of the most active agents, that is, the platinum compounds cisplatin and carboplatin and gemcitabine, alone or in combination, we performed colony formation assays in the breast cancer cell lines MDA-MB231 and HS578T. As can be seen in Figure 2a, the combination of the platinum agents and gemcitabine with rabusertib had more effect than each agent given alone. Finally, we conducted Matrigel invasion studies to explore the effect of rabusertib with platinum agents and gemcitabine on 3D invading structures growth. Again, the combination displayed more activity than each compound as a single agent for the two cell lines studied (Figure 2b). This set of experiments confirms the effect of the combination of these agents on proliferation, invasion, and long-term survival in basal-like cancer cell lines.

### 2.3. Combination of Platinum Agents and Gemcitabine with Rabusertib Induces Cell Death through a Caspase-Dependent Mechanism

Next, we aimed to explore the effect of the combination of platinum compounds and gemcitabine with rabusertib on the cell cycle. To do so, MDA-MB-231 and HS578T were exposed to each agent alone or combined with rabusertib. Single treatment with rabusertib slightly increased G1 for both cell lines; meanwhile platinum compounds increased G2/M, mainly in MDA-MB-231. Combination of the platinum compounds with rabusertib did not exhibit any obvious effect for either cell line (Figure S6). On the other hand, although exposure to gemcitabine slightly augmented S, combination with the CHK1 inhibitor did not clearly modify the cell cycle pattern (Figure S6).

**Figure 3:**
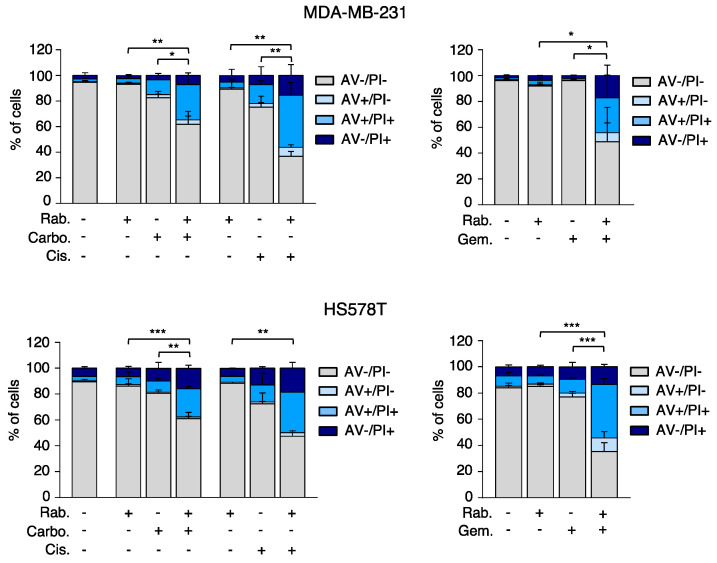
The combination of rabusertib with carboplatin, cisplatin or gemcitabine promotes cell death. Cells were seeded and then exposed at the same doses of rabusertib, cisplatin, carboplatin and gemcitabine indicated in Figure 2, but for 72 h. Then, the percentage of Annexin V +/− and PI +/− cells was determined by flow cytometry. Statistics for single against double combination are shown. *p* < 0.05 *, *p* < 0.01 **, *p* < 0.001 ***.

Even though we did not find a clear impact on cell cycle, we hypothesized that genomic instability might be increased with the combination of both agents due to DNA damage, therefore, making the cells enter the apoptotic process. To evaluate the potential induction of cell death, we treated the breast cancer cell lines with the agents alone or in combination with the CHK1 inhibitor, and after 72 h of treatment, exposed them to an Annexin V/PI solution to allow cell death determination by flow cytometry. As can be seen in Figure 3, treatment with the combination intensely increased apoptosis in the two cell lines tested, especially for the combination with gemcitabine, and showed statistical significance for all combinations. Still, the combination of rabusertib with DNA agents did not show a toxic effect on noncancerous breast tissue-derived cells (Figure S7).

**Figure 4:**
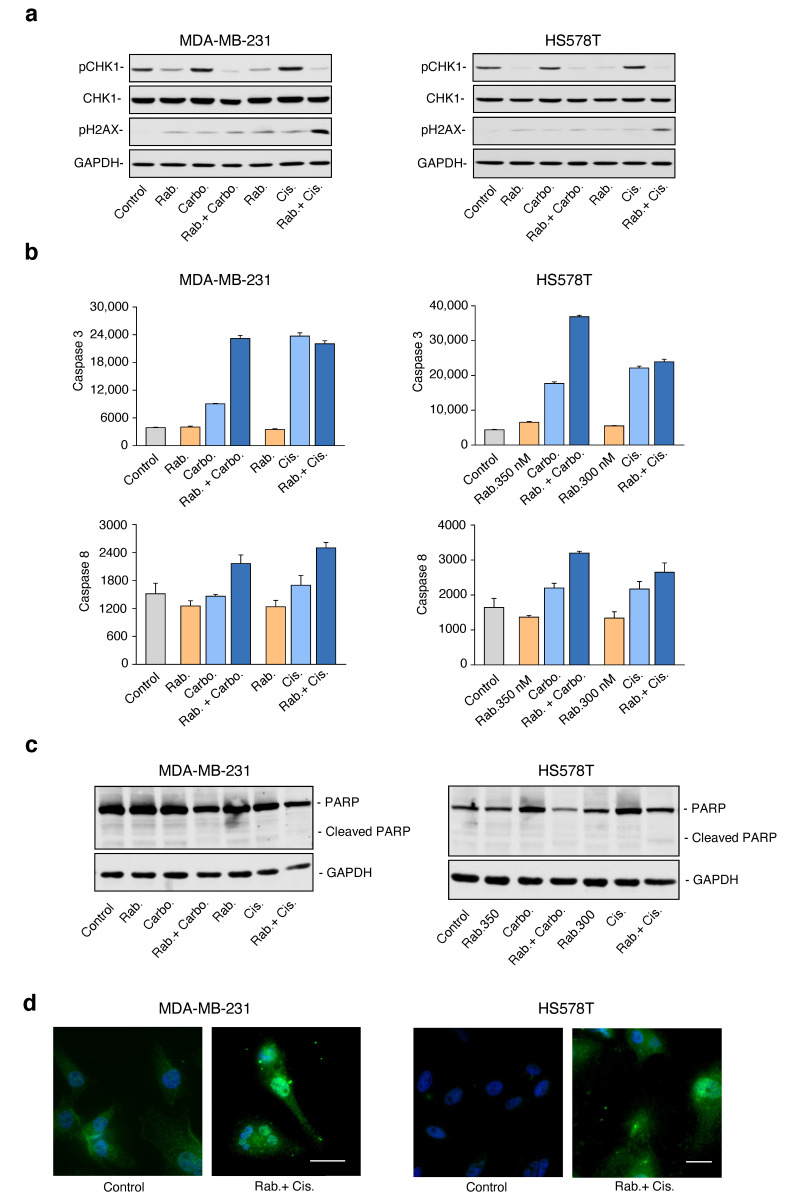
The combination of rabusertib with carboplatin and cisplatin increases DNA damage and the activity of caspases implicated in apoptosis. (**a**–**c**) Cells were treated as in Figure 3. (**a**) Then, levels of pCHK1, CHK1, and pH2AX were evaluated by Western blot as described in Section 4. Levels of GAPDH were used as loading control. (**b**) Cells were lysed, and caspase reaction buffer was added to cell lysates as described in Section 4. Signals were measured in a fluorescent reader (BioTek). (**c**) After drug exposure, proteins extracts were loaded in a SDS-PAGE and PARP protein bands were visualized. Levels of GAPDH were used as loading control. (**d**) Fluorescence microscopy was used to obtain images of cleaved caspase 3 immunoreactivity (green) and DNA staining (DAPI, blue). Representative images showing cleaved caspase 3 expression in non-treated cells (control) and rabusertib + cisplatin (combination) in MDA-MB-231 cells (left) and HS578T cells (right) are shown. Scale bar = 50 μM.

To study the biochemical effect of the treatments, we used Western-blotting to analyze several proteins involved in the activation of DNA damage repair pathways. Rabusertib efficiently inhibited the phosphorylation of CHK1 in both cell lines tested (Figure 4a). In contrast, platinum compounds induced DNA damage, which was measured by the increase in pH2AX, and the subsequent increase in the phosphorylation of CHK1, as a consequence of the activation of the DNA response machinery (Figure 4a). Of note, the combination of both agents increased pH2AX in all cell lines, thus supporting a high genotoxic effect under these conditions (Figure 4a). 

Next, we explored the mechanisms associated with the induction of apoptosis. A marked increase in Caspase 3 and 8 activities was observed in response to the combinations for both cell lines (Figure 4b). The biochemical evaluation also showed that full-length PARP levels were altered after treatment with the combination, while no clear PARP cleavage was detected (Figure 4c). However, a strong increase in the cleaved form of caspase 3 was observed by immunofluorescence upon exposure to the combination of the drugs (Figure 4d), further supporting the activation of the caspase cascade in response to the combination.

Taken together, this data suggests that inhibition of CHK1 hampers cell cycle arrest in the presence of DNA damage, allowing cells to progress, therefore increasing the genomic instability. Also, these results demonstrate that the induction of apoptosis by the combination of platinum agents with rabusertib was mainly mediated by activation of caspases. 

### 2.4. Inhibition of CHK1 Reverts Resistance to Platinum Compounds

**Figure 5:**
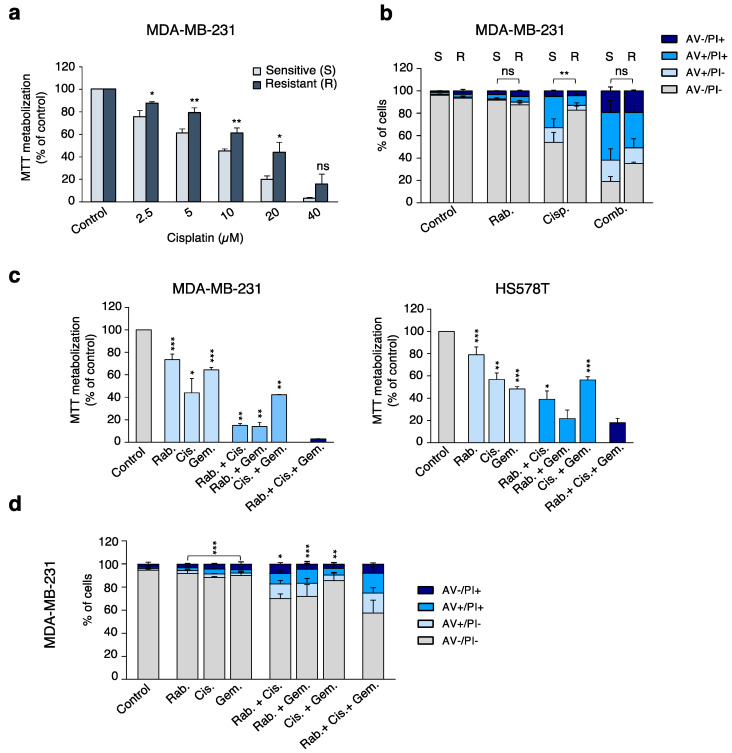
Rabusertib enhances cell death in cisplatin resistant cells and impacts on cell proliferation when combined with cisplatin and gemcitabine. (**a**) Resistance to cisplatin was corroborated by MTT assay where cells were treated at the indicated doses for 72 h. (**b**) Naïve (S) or cisplatin-resistant (R) MDA-MB-231 cells were treated with rabusertib (Rab. 500 nM) and cisplatin (Cis. 10 μM) for 72 h. Then, the percentage of Annexin V +/− and V +/− cells was determined by flow cytometry. Statistics showed differences between S and R. (**c**,**d**) TNBC cell lines were treated with CHK1 inhibitor (Rab. 200 nM for MDA-MB-231 and 300 nM for HS578T), cisplatin (Cis. 5 μM), gemcitabine (Gem. 2nM for MDA-MB-231 and 200 nM for HS578T), or their combinations for 72 h. (**c**) Then, metabolization of MTT in viable cells was determined by spectrophotometry or (**d**) the percentage of Annexin V +/− and PI +/− cells was evaluated by flow cytometry. Statistics of single and double treatments against triple combination are shown. ns > 0.05, *p* < 0.05 *, *p* < 0.01 **, *p* < 0.001 ***.

To evaluate the effect of rabusertib on the resistance to platinum compounds, we took advantage of a cisplatin-resistant model generated in our laboratory and derived from the parental cell line MDA-MB-231, MDA-MB231R, as described in material and methods. Resistance to cisplatin was corroborated before the beginning of the experiments (Figure 5a). Both parental and cisplatin-resistant cell lines were then treated with cisplatin, rabusertib, or the combination of both agents. As expected, treatment with cisplatin alone did not have a marked impact on cell death in the resistant model but was very active in the sensitive parental one (Figure 5b). Treatment with CHK1 inhibitor alone did not produce a significant effect in any of the models. Remarkably, addition of rabusertib to cisplatin noticeably increased cell death in both cell lines, including the resistant ones (Figure 5b). These data demonstrate that resistance to cisplatin can be overcome by inhibition of the CHK1 kinase. 

### 2.5. CHK1 Inhibition Enhances the Effect of Standard-of-Care Chemotherapies

Clinical development of most new compounds is based on their combination with approved chemotherapies. In this context, we decided to explore the effect of the CHK1 inhibitor rabusertib in combination with the two most active chemotherapies shown in Figure 1b, cisplatin and gemcitabine. As can be seen in Figure 5c, the triple combination had a profound effect compared with single agent or double combinations in basal-like breast cancer cell lines, leading to a higher impact on cell death (Figure 5d).

## 3. Discussion

Selection of synergistic combinations based on synthetic lethality interactions that pose no toxicity issues is crucial in drug development [7,22]. In this context, the identification of agents that regulate DDR and can augment the effect of DNA-damaging agents has important implications in the clinical setting. 

In this work, we proposed a combination of agents that are synergistic in basal-like breast cancer, including TNBC, a disease where therapeutic options are limited and there is a clear need to identify new treatments. Of note, this combination includes approved chemotherapies for the treatment of these patients or agents that although not approved are included in clinical guidelines, so their implementation in the clinical setting will not be a limitation.

Basal-like breast cancers commonly carry mutations in genes linked with DNA repair mechanisms like BRCA1, BRCA2, or RAD51. This has led to the development of strategies that act on DNA repair mechanisms, some of which have been quickly translated into the clinical setting, as is the case for the PARP inhibitor olaparib in breast cancer [23,24]. Despite these recent advances, most therapeutic options for this disease are still restricted to combinations of chemotherapies [23]. 

As we have demonstrated in this article, the combination of DNA-damaging agents such as platinum compounds or gemcitabine, but also topotecan and doxorubicin, with CHK1 inhibitors has a synergistic effect on basal-like breast cancer cells that is not observed when combined with agents targeting mitosis, for example, taxotere, eribulin, or vinorelbine. Moreover, the combination of the platinum compound, cisplatin and the CHK1 inhibitor, rabusertib did not have a toxic effect on non-tumorigenic breast tissue-derived cells, which suggests that the high efficacy of the combination of CHK1 inhibitors and DNA-damaging agents is restricted to cancer cells. A similar antiproliferative effect was observed for PARP inhibitors, reinforcing the concept that inhibition of CHK1 allows cells to enter apoptosis when a relevant genetic instability is present, in this case due to alterations of DNA repair mechanisms or an increase in DNA damage [25]. Therefore, we found that agents that induced DNA damage, like gemcitabine or platinum compounds or to a less extent doxorubicin and topotecan, were synergistic in breast cancer cell lines. This was particularly evident for platinum compounds. Results in line with these findings have been observed in other tumor types such as bladder, pancreatic, or colon cancer [20,22]. However, we recognize that this effect was not observed for some combinations and cell lines, which might be due to the heterogeneity of these cell lines. For instance, the combination of the platinum agent carboplatin and the CHK1 inhibitor in the breast cancer cell line HCC3153. This fact indicates the heterogeneous nature of cancer, which is reiterated, as recently demonstrated, in cell lines [26]. Importantly, our results show that the CHK1 inhibitor, rabusertib was able to overcome resistance to cisplatin in a resistant model of breast cancer, suggesting its potential use in combination in patients presenting with resistance to platinum agents [27]. 

In cells that are exposed to DNA damaging-agents, activation of CHK1 induces cell cycle arrest and the initiation of DNA repair mechanisms [14,15]. Inhibition of CHK1 permits cell cycle progression without repairing lesions at a DNA level, increasing genomic instability and finally inducing apoptosis [14,15]. Of note, several CHK1 inhibitors are currently in the early stage of clinical development [13,22]. 

Given the fact that the combination of CHK1 inhibitors with platinum compounds was particularly synergistic, we decided to explore the mechanism of action of this combination, focusing on the biochemical analyses of combinations with cisplatin. The association with cisplatin did not have a clear effect on the pattern of the cell cycle. This finding can be explained as cells can be at different phases of the cell cycle, which limits the observation of any clear action on the cell cycle. This pleotropic effect has been described on many occasions when agents are administered in combination, and, therefore, it is not surprising [28]. However, this observation does not limit the anti-tumoral effect observed. In line with this, the combination of both agents produced a marked induction of apoptosis. Experiments evaluating caspase cleavage and activity demonstrated that the induction of apoptosis was accompanied by caspase cascade activation. 

As expected, administration of platinum compounds induced DNA damage measured by pH2AX, although the effect of each platinum agent can differ between cell lines due to their different mode of action. A similar finding was observed with the CHK1 inhibitor, as it prevents cells from entering the arrest phase, permitting cell cycle progression and, therefore, increasing the genetic instability. The combination of both agents augmented the presence of pH2AX, and demonstrated a clear increase in DNA damage. 

We also explored the combination of agents that have a clear translation to the clinical setting, including double combinations with chemotherapies such as cisplatin and gemcitabine. We observed that these triple combinations were more effective than double or single agents given alone. This finding has important implications for the clinical setting, as cisplatin and gemcitabine are standard treatments in non-small cell lung cancer, ovarian cancer and triple negative breast cancer. 

Our study has limitations, in particular it is important to recognize that the evaluation of synergistic interactions in cell lines only constitutes an initial step in the development of novel compounds. Only when explored in humans, where other factors such as the immune system or the tumor microenvironment play a role, can the combinational effect be confirmed [29,30]. However, some examples that use in vitro models to evaluate synergism have confirmed their effect later on when evaluated in humans, and therefore these findings were scientifically relevant [31]. In addition, as mentioned, this work has the intrinsic limitation, which is also a strength, of using cell lines that have a wide range of heterogeneity [23]. 

**Figure 6:**
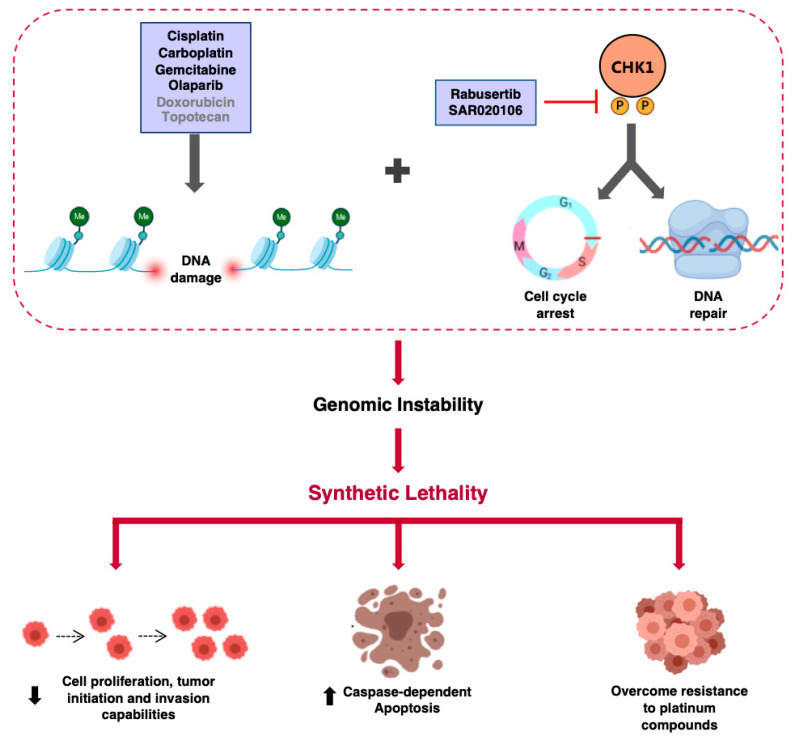
Diagram of the mechanism of action of CHK1 inhibitors in combination with platinum compounds. DNA damage agents induce phosphorylation of CHK1, leading to cell cycle arrest and the activation of the DNA repair machinery. Combination of CHK1 inhibitors with DNA-damaging compounds increases genomic instability, which results in a decrease of cell proliferation and invasion capabilities, eventually unraveling a potent apoptotic response, even in platinum-resistant cells.

The data presented here suggests several interesting therapeutic options. On one hand, the finding that DDR inhibitors targeting CHK1 potentiate the action of DNA-damaging agents that are used in the clinical setting provides an important clue for the clinical development of CHK1 inhibitors. Furthermore, the reversal of developed resistance to platinum agents observed when combined with CHK1 inhibitors may have therapeutic relevance for those patients being treated with platinum derivates (Figure 6).

## 4. Material and Methods

### 4.1. Cell Lines and Cultures

Breast cancer cell lines were kindly provided by Dr. A. Pandiella. Cell lines were authenticated by STR profiling. They were grown in DMEM (MDA-MB-231, HS578T, and BT549) or RPMI (HCC3153). All media was supplemented with 10% fetal bovine serum (FBS), 100 U/mL penicillin, 100 μg/mL streptomycin and 2 mM L-glutamine. The non-transformed epithelial cell line MCF10A, derived from a noncancerous mammary gland, was purchased from Elabscience^®^ and maintained in DMEM/F12 supplemented with 5% horse serum, 20 ng/mL EGF, 0.5 μg/mL hydrocortisone, 10 μg/mL insulin, 1% non-essential amino acids plus the abovementioned antibiotics. The main characteristics of all these cell lines are summarized in the following Table 1. 

Normal fibroblasts, CRL-2072, derived from breast skin, were kindly provided by the Translational Research Unit of the Ciudad Real University Hospital, Spain, and were grown in DMEM with 10% FBS, 100 U/mL penicillin and 100 μg/mL streptomycin. Cell culture media and supplements were obtained from Sigma Aldrich (St. Louis, MO, USA).

Cisplatin-resistant breast cancer cells (MDA-MB-231R) were generated by exposure to increasing concentrations of cisplatin (from IC30 to IC70) for 5–6 months (EMGM and MNC).

All cell lines were maintained at 37 °C in a 5% CO_2_ atmosphere.

### 4.2. MTT and Synergistic Effect of Rabusertib and Chemotherapic Agents

Rabusertib, docetaxel, vinorelbine, gemcitabine, topotecan and olaparib were purchased from Selleckchem (Munich, Germany). Carboplatin and Cisplatin were purchased from Accord Healthcare (Middlesex, UK). Eribulin was from Eisai Inc. (Tokyo, Japan) and doxorubicin from Pfizer GEP, SL (NY, NY, USA).

Dose-response and drug combination studies were performed using MTT screening assays. Thus, cells were seeded into 48-multiwell plates (1 × 10^4^ cells per well), and 24 h later, they were treated either with single agents or a combination of them (IC50 or lower for the synergy studies). MTT (0.5 mg/mL) was added to the wells 72 h later and then they were incubated for 1 h at 37 °C. Then, the MTT was removed and DMSO was added to solubilize the formed crystals. Last, absorbance values were measured at 555 nm (555–690) in a multiwell plate reader (BMG Labtech, Ortenberg, Germany).

Results were plotted as the mean values of three independent experiments. Drugs interactions were calculated by determining combinational index (CI), based on the algorithm reported by Chou and Talalay, using Calcusyn Version 2.0 software (Biosoft, Ferguson, MO, USA). CI < 1 = synergistic effect; CI = 1 = additive effect; CI >1 = antagonistic effect.

### 4.3. Clonogenic Assays and Matrigel Embedded 3D Cultures

For clonogenic experiments, cells were seeded in a 60 mm plates (5 × 10^5^ cells per plate) and treated the following day with the indicated doses of the drugs. Twenty-four hours later, cells were tripsinized, counted and resuspended in complete growth medium to perform serial dilutions (1/10). We selected dilutions 3 and 4 to be seeded in triplicate in 6-multiwell plates and maintained for 10 days, when the number of colonies was counted. 

For 3D culture studies, cells were seeded (5 × 10^3^ cells per well) in 48-multiwell plates containing an underlying layer of Matrigel, which was pre-incubated at 37 °C during 30 min. The following day, cells were treated with the indicated doses of the drugs. The diameter of invading 3D colonies was monitored daily under a light microscope for 3 days.

### 4.4. Cell Cycle and Apoptosis Studies

For cell cycle analysis, cells (2.5 × 10^5^ cells in 100 mm plates) were treated with the drugs at the indicated doses for 24 h. Then, cells were harvested by trypsinization, washed with cold PBS and fixed with 70% ethanol for 30 min. After centrifugation, cell pellets were washed with PBS and incubated in the dark with Propidium Iodide (PI)/RNAse staining solution (Immunostep S.L., Salamanca, Spain) for 1 h at 4 °C.

For the apoptosis assays, cells were treated as described above but for 72 h. Then, supernatants and cells were collected and washed twice with cold-PBS. For the analysis, cells were stained with Annexin V-DT-634 (Immunostep S.L., Salamanca, Spain) and 3 μL of PI (10 mg/mL) in 1× Binding Buffer (10 mM HEPES, pH 7.4, 140 mM NaOH, 2.5 mM CaCl_2_) for 1 h at room temperature in the dark. Stained cells were immediately analyzed using a FACSCanto II flow cytometer (BD Biosciences, San Jose, CA, USA). Early (Annexin V-positive, PI-negative) and late (Annexin V-positive and PI-positive) apoptotic cells were included in the determination of cell death.

### 4.5. Caspase Activity Assays

Cells (5 × 10^5^) were treated with rabusertib, carboplatin, cisplatin at the indicated doses for 72 h. Then, they were lysed in ice-cold apoptosis lysis buffer (20 mM Tris, 140 mM NaCl, 10 mM EDTA, 10% glycerol, 1% NP40, pH 7.0), supplemented with protease inhibitors (Sigma Aldrich, St. Louis, MO, USA) and the protein concentration was determined using Pierce BCA (Bicinchoninic acid) protein assay (Thermo Fisher Scientific, Waltham, MA, USA). The final volume of the lysates was taken to 100 μL by 1× Caspase buffer (25 mM HEPES pH 7.4, 150 mM NaCl, 1 mM EDTA, 0.1% CHAPS, 10% sucrose). Then, 100 μL of Caspase reaction buffer 2 × (50 mM HEPES pH 7.4, 300 mM NaCl, 2 mM EDTA, 0.2% CHAPS, 20% sucrose, 20 mM DTT and 10 μM of fluorescently-labelled caspase substrate Ac-IETD-AFC or Ac-DEVD-AFC) was added to each well containing cell lysates. The plate was shaken to mix the solution and incubated at 37 °C for 1 h. Signals were measured at 400/505 nm in a fluorescent reader (BioTek, Winooski, VT, USA).

### 4.6. Western Blotting

MDA-MB-231 and HS578T were treated with rabusertib, carboplatin, and cisplatin, as described in Section 4.4, for 24 h. Then, cells were lysed and the protein concentration was determined. 50 μg of total protein was loaded in an SDS-PAGE electrophoresis system. Blots were blocked in Tris-buffered saline (TBS)-5% milk and incubated overnight with the following primary human antibodies. The anti-GAPDH and anti-PARP antibodies were purchased from Santa Cruz Biotechnology (Santa Cruz, CA, USA). The anti-pH2AX, anti-pCHK1 (Ser 296) and anti-CHK1 antibodies were from Cell Signaling Technologies (Beverly, MA, USA). Horseradish peroxidase conjugates of anti-rabbit and anti-mouse immunoglobulin G (IgG) were from Bio-Rad Laboratories (Hercules, CA, USA). Protein bands were visualized by a luminal-based detection system with p-iodophenol enhancement.

### 4.7. Immunofluorescence

Cells were grown on glass coverslips and treated with the indicated drugs for 72 h. Then, coverslips were washed and cells were fixed in 4% paraformaldehyde for 10 min at room temperature, rinsed twice with PBS, and blocked in PBS containing 0.1% Triton X-100 and 4% BSA for 1 h, and subsequently incubated overnight at 4 °C with anti-cleaved caspase 3 antibody (1:200, Cell Signaling Technology, Danvers, MA, USA). The coverslips were washed and incubated with an anti-rabbit Alexa Fluor 488 (1:1000) antibody for 60 min. DAPI (300 nM) was added for 3 min and the coverslips were washed again twice with PBS before mounting. Fluorescence imaging was performed using a Nikon epifluorescence inverted microscope. The excitation laser power percentage, time of exposition and camera binning were not changed among the different experiments in order to compare the cleaved-caspase 3 expression between conditions.

### 4.8. Statistical Analysis

One-way ANOVA and the T-Student test was used to determine significant statistical differences (*p* < 0.05 *, *p* < 0.01 **, *p* < 0.001 ***). 

To analyze the synergy studies, the mean CI for each combination was compared with the value of CI = 1 to check if the values were statistically significant. 

## Figures and Tables

**Table 1:** Characteristics of breast epithelial cell lines. Information obtained from the ATCC.

Cell Lines	Origin		Receptor Expression				Mutations
	Tissue	Disease	EGFR	ER	PR	HER2	
MDA-MB-231	Mammary gland/breast; derived from metastatic site: pleural effusion	Epithelial adenocarcinoma	Positive	Negative	Negative	Positive (low expression)	BRAF, CD79A, CRTC3, NF2, PCSK7, PDGFRA, TP53
BT549	Mammary gland/breast	Epithelial ductal carcinoma	Positive	Negative	Negative	Negative	PTEN, TP53, RB1
HS578T	Mammary gland/breast	Epithelial carcinoma	Positive	Negative	Negative	Negative	HNF1A, NF1, PIK3R1, TP53
HCC1353	Mammary gland/breast	Ductal carcinoma	Positive	Negative	Negative	Negative	BRCA1
MCF10A	Mammary gland/breast	Fibrocystic disease	Positive	Negative	Negative	Negative	-

## Supplementary Materials

The following are available online at https://www.mdpi.com/1422-0067/21/23/9034/s1.
