# Supplementary material for: Checkpoint Kinase 1 Pharmacological Inhibition Synergizes with DNA-Damaging Agents and Overcomes Platinum Resistance in Basal-Like Breast Cancer
Source: Int J Mol Sci. 2020 Nov 27;21(23):9034. doi: 10.3390/ijms21239034 (PMC7730833; doi:10.3390/ijms21239034)
Supplement: Supplementary file 1 [file ijms-21-09034-s001.pdf]

Legends to supplementary figures

Figure S1. IC50 values for all cell lines. (a) IC50 values for all chemotherapy agents tested. **(b)** IC50 to the Chk1 inhibitor rabusertib. All values are expressed in the nanomolar scale (nM).

Figure S2. Lack of synergistic effect between Chk1 inhibitor and cisplatin in noncancerous breast cell lines. (a) MCF10A and CRL-2072 cells were treated with rabusertib and cisplatin, alone or in combination, for 72 h at the indicated doses. Then, MTT was added to cells and solubilized with DMSO. Absorbance values were measured at 562 nm in a multiwell plate reader (BMG labtech). (**b)** Combination indexes for rabusertib and cisplatin for both cell lines were obtained using CalcuSyn program, plotted and compared with the results obtained for the basal-like cancer line MDA-MB-231. $p \geq 0.05$ ns, $p<0.001$ ***

Figure S3. Synergistic anti-proliferative effect between Chk1 inhibitor and olaparib in breast cancer cell lines. (a) Cell lines were treated with rabusertib and olaparib for 72 h at the indicated doses. Then, MTT was added to cells and solubilized with DMSO. Absorbance values were measured at 562 nm in a multiwell plate reader (BMG labtech). (**b)** Combination indexes for rabusertib and olaparib were obtained using CalcuSyn program and plotted. $p<0.05$ *, $p<0.01$ **, $p<0.001$ ***

Figure S4. Combination indexes for rabusertib and the chemotherapeutic agents doxorubicin, topotecan, vinorelbine, docetaxel, and eribulin. (a) Cell lines were treated with increasing doses of rabusertib, doxorubicin or topotecan for 72h. Then, viable cells were detected by MTT assay and combination indexes were obtained using CalcuSyn program and plotted. (**b)** Same cell lines were treated with increasing doses of rabusertib and vinorelbine, docetaxel or eribulin.

Figure S5. SAR020106, another Chk1 inhibitor, also shows a synergistic effect when combined with platinum compounds. (a) Cell lines were treated with SAR020106 and cisplatin for 72h at the indicated doses. Then, percentage of viable cells was determined using MTT assay and combination indexes values were obtained using CalcuSyn. (**b)** Combination indexes for Chk1 inhibitor and carboplatin. $p<0.05$ *, $p<0.01$ **, $p<0.001$ ***

Figure S6. The combination of rabusertib with carboplatin, cisplatin or gemcitabine produces a pleiotropic effect on cell cycle. Cells were treated with rabusertib (Rab. 300 nM or 350 nM for cisplatin or carboplatin sample sets, respectively) and carboplatin (Carbo. 35µM) or cisplatin (Cis. 7µM) for 24 h. For gemcitabine sample sets, cells were treated with rabusertib (Rab. 500nM or 200nM, for MDA-MB-231 or HS578, respectively) and gemcitabine (Gem. 5nM or Gem. 300 nM, for MDA-MB-231 or HS578, respectively) for 24 h. Then, cell cycle progression was evaluated by flow cytometry.

Figure S7. The combination of rabusertib with cisplatin does not increase cell death in normal breast cell lines. MCF10A and CRL-2072 cells were seeded and, then, exposed to rabusertib (Rab. 300nM) and cisplatin (Cis. 7µM), alone or in combination, for 72 hours. Then, percentage of Annexin V+/- and PI +/- cells was determined by flow cytometry. Statistics of single against double combination are shown. $p \geq 0.05$ ns, $p<0.05$ *, $p<0.01$ **.

a

	MDA-MB-231	HS578T	BT549	HCC3153
Carboplatin	50000	50000	47500	50000
Cisplatin	6000	7000	2000	13000
Doxorubicin	23	125	23	200
Topotecan	100	5000	100	1000
Gemcitabine	7,5	1000	10	500
Olaparib	10	20	10	100
Docetaxel	0,2	0,25	0,6	0,8
Eribulin	1	3	3	5
Vinorelbine	1	1	0,7	5

b

	MDA-MB-231	HS578T	BT549	HCC3153
Rabusertib	400	600	300	500

Figure S1

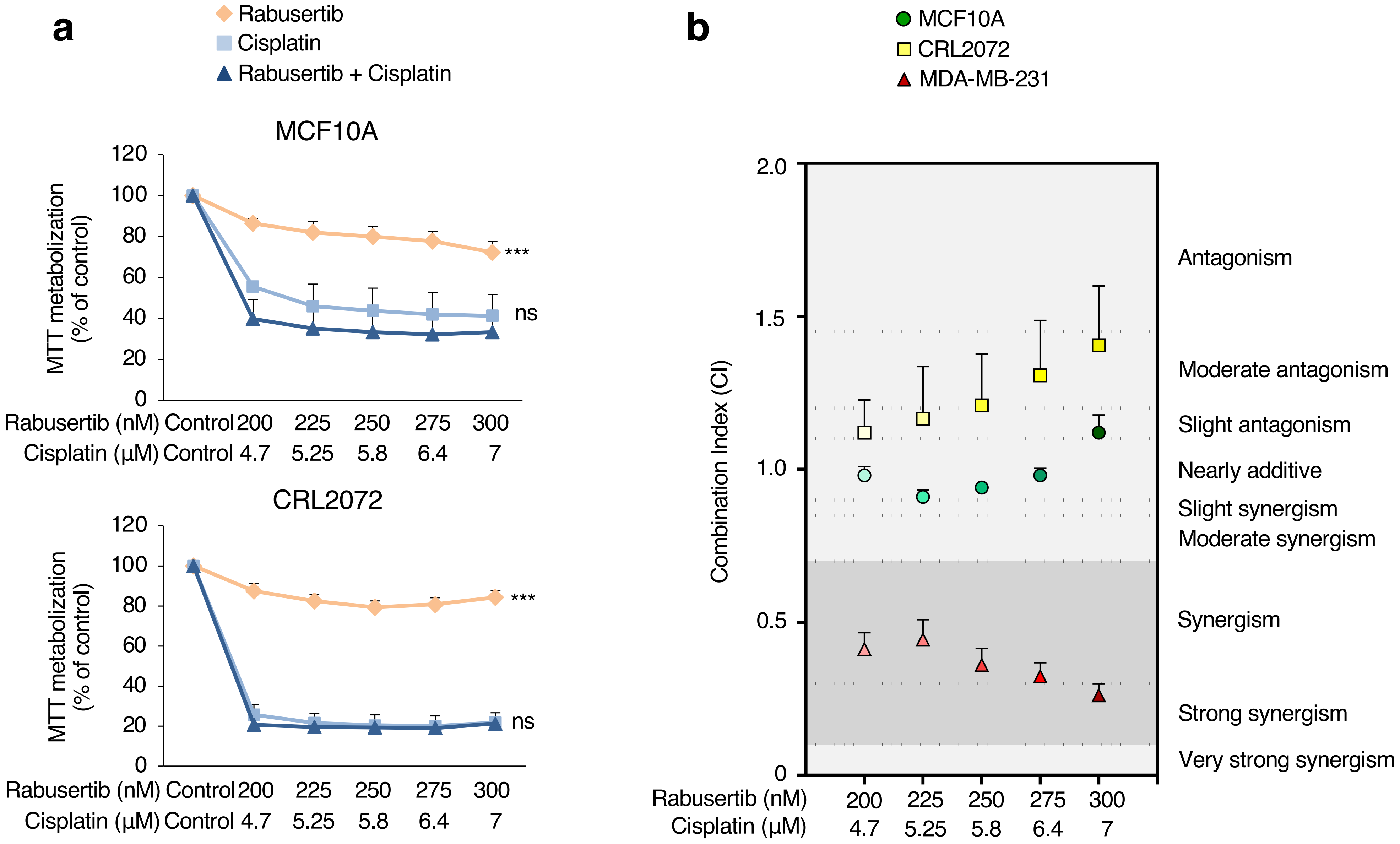
a
Rabusertib
Cisplatin
Rabusertib + Cisplatin
MCF10A
MTT metabolization (% of control)
120
100
80
60
40
20
0
***
ns
Rabusertib (nM) Control 200 225 250 275 300
Cisplatin (µM) Control 4.7 5.25 5.8 6.4 7
CRL2072
MTT metabolization (% of control)
120
100
80
60
40
20
0
***
ns
Rabusertib (nM) Control 200 225 250 275 300
Cisplatin (µM) Control 4.7 5.25 5.8 6.4 7
b
MCF10A
CRL2072
MDA-MB-231
Combination Index (CI)
2.0
1.5
1.0
0.5
0
Antagonism
Moderate antagonism
Slight antagonism
Nearly additive
Slight synergism
Moderate synergism
Synergism
Strong synergism
Very strong synergism
Rabusertib (nM) 200 225 250 275 300
Cisplatin (µM) 4.7 5.25 5.8 6.4 7

Figure S2

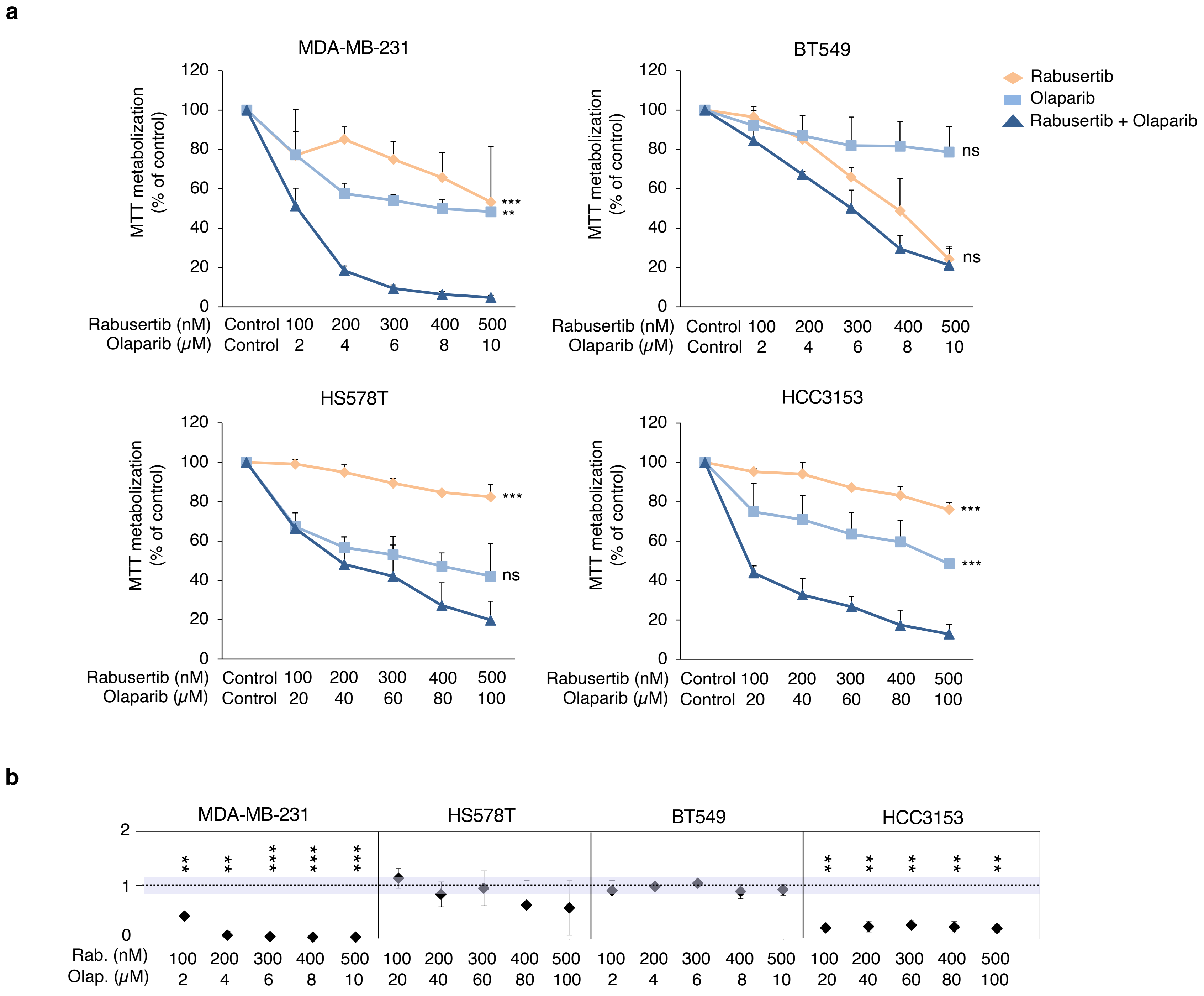

a
MDA-MB-231
BT549
HS578T
HCC3153
MTT metabolization (% of control)
Rabusertib
Olaparib
Rabusertib + Olaparib
Rabusertib (nM) Control 100 200 300 400 500
Olaparib (μM) Control 2 4 6 8 10
Rabusertib (nM) Control 100 200 300 400 500
Olaparib (μM) Control 20 40 60 80 100
***
**
ns
b
Rab. (nM)
Olap. (μM)

Figure S3

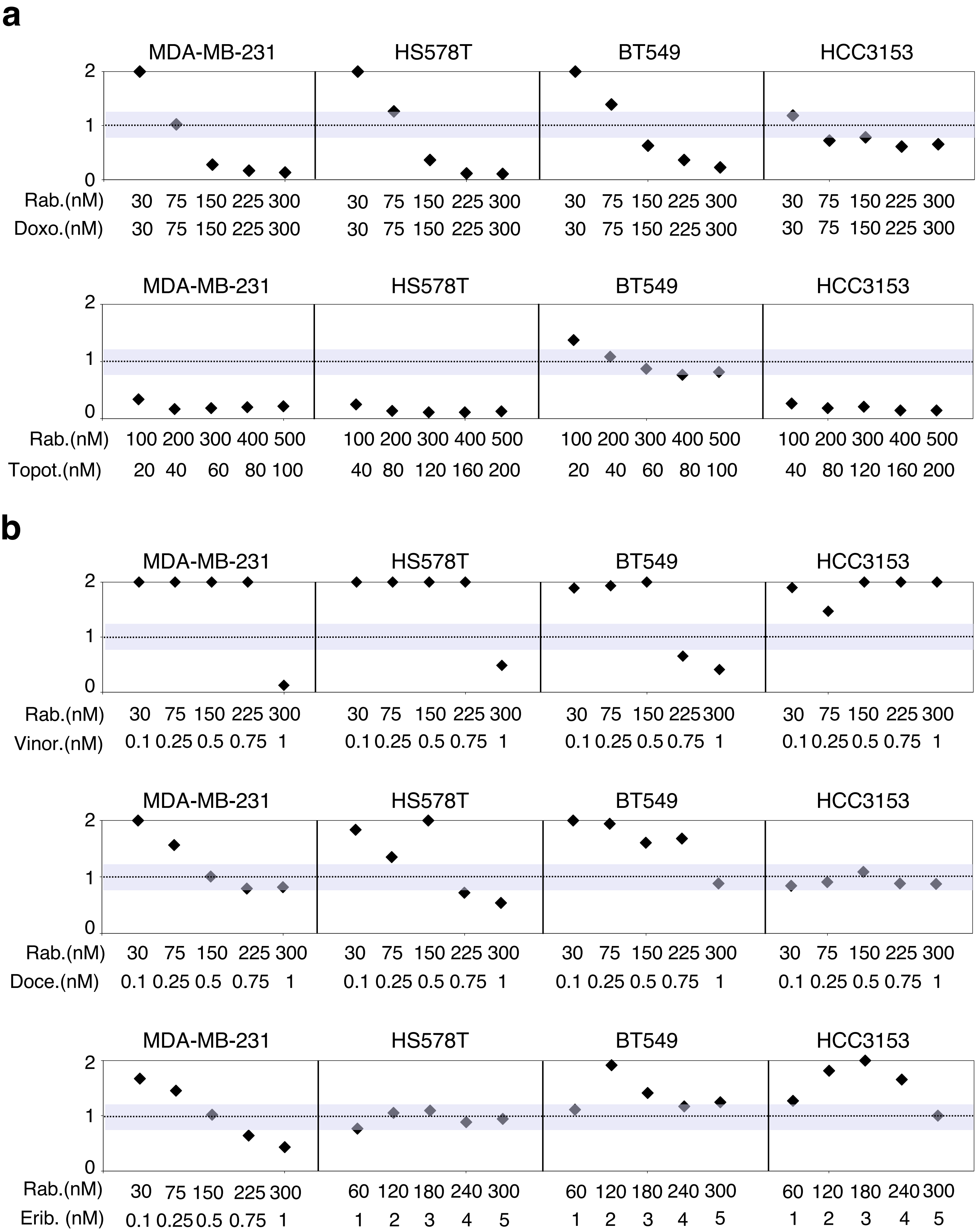
a
MDA-MB-231
HS578T
BT549
HCC3153
2
1
0
Rab.(nM) 30 75 150 225 300
Doxo.(nM) 30 75 150 225 300
Rab.(nM) 100 200 300 400 500
Topot.(nM) 20 40 60 80 100
40 80 120 160 200
b
Rab.(nM) 30 75 150 225 300
Vinor.(nM) 0.1 0.25 0.5 0.75 1
Doce.(nM) 0.1 0.25 0.5 0.75 1
Rab.(nM) 60 120 180 240 300
Erib. (nM) 0.1 0.25 0.5 0.75 1
1 2 3 4 5

Figure S4

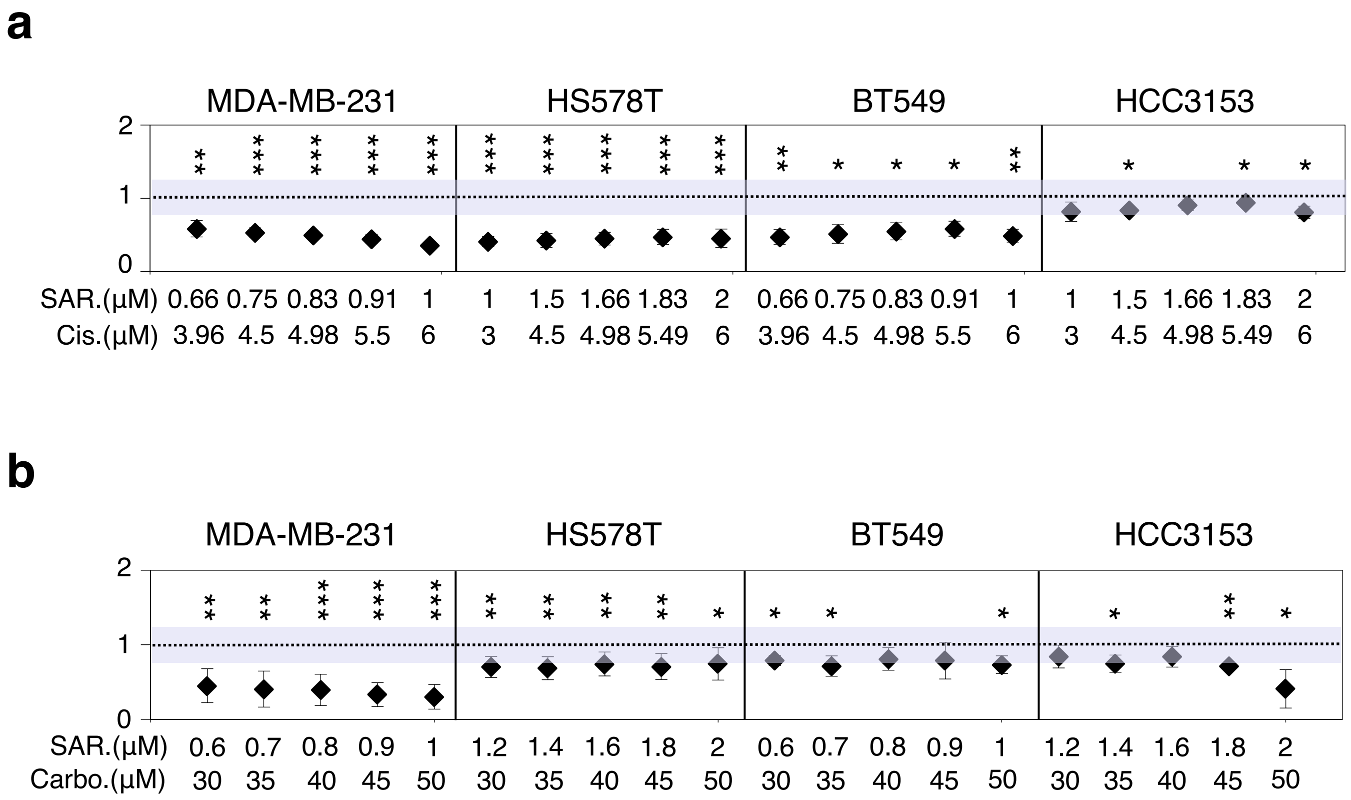
a
MDA-MB-231
HS578T
BT549
HCC3153
2
1
0
SAR.(µM) 0.66 0.75 0.83 0.91 1 1 1.5 1.66 1.83 2 0.66 0.75 0.83 0.91 1 1 1.5 1.66 1.83 2
Cis.(µM) 3.96 4.5 4.98 5.5 6 3 4.5 4.98 5.49 6 3.96 4.5 4.98 5.5 6 3 4.5 4.98 5.49 6
b
MDA-MB-231
HS578T
BT549
HCC3153
2
1
0
SAR.(µM) 0.6 0.7 0.8 0.9 1 1.2 1.4 1.6 1.8 2 0.6 0.7 0.8 0.9 1 1.2 1.4 1.6 1.8 2
Carbo.(µM) 30 35 40 45 50 30 35 40 45 50 30 35 40 45 50 30 35 40 45 50

Figure S5

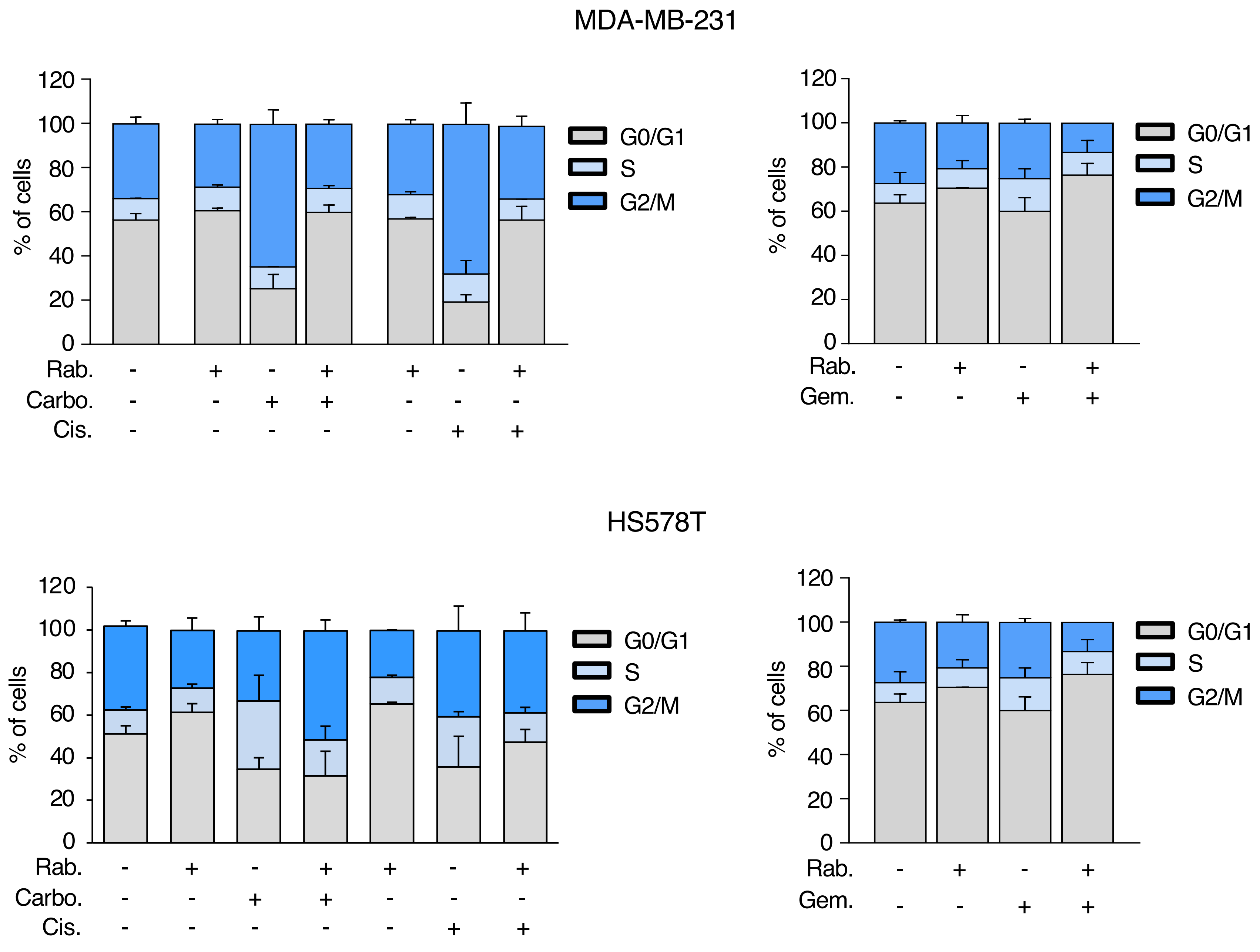
MDA-MB-231
120
100
80
60
40
20
0
% of cells
G0/G1
S
G2/M
Rab.
Carbo.
Cis.
Gem.
HS578T

Figure S6

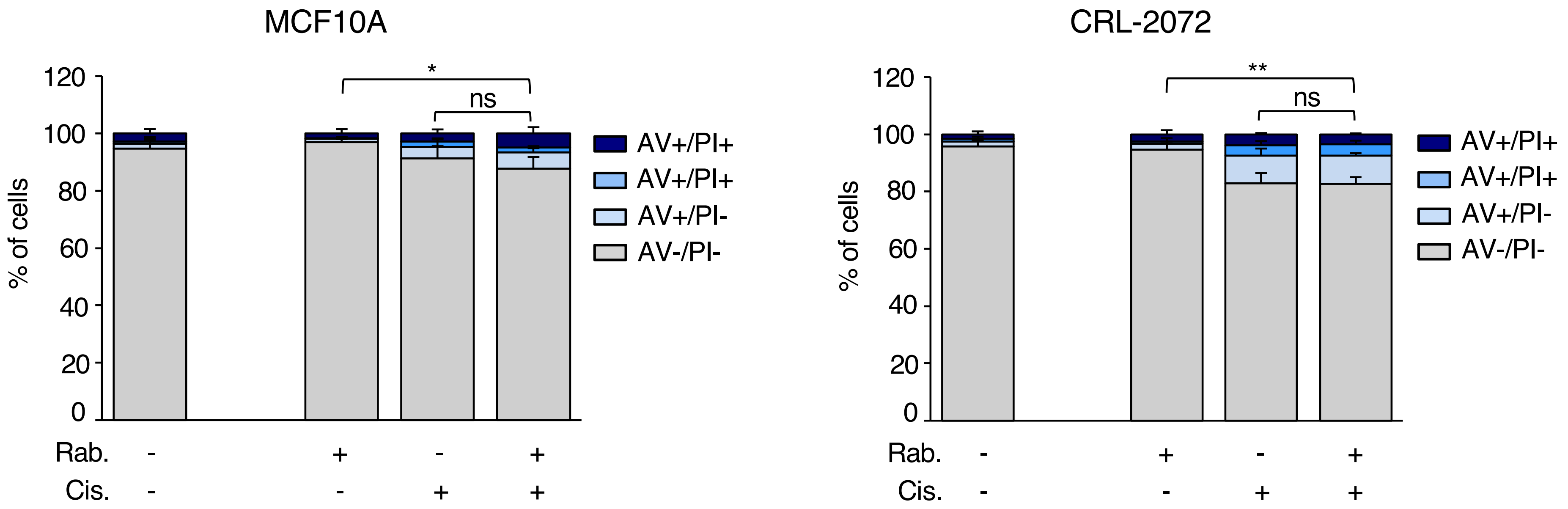
MCF10A
CRL-2072
120
100
80
60
40
20
0
% of cells
*
**
ns
AV+/PI+
AV+/PI+
AV+/PI-
AV-/PI-
Rab. - + - +
Cis. - - + +

Figure S7
